# Role of the gut microbiome in three major psychiatric disorders

**DOI:** 10.1017/S0033291722000897

**Published:** 2022-05

**Authors:** Jenny Borkent, Magdalini Ioannou, Jon D. Laman, Bartholomeus C. M. Haarman, Iris E. C. Sommer

**Affiliations:** 1 Department of Biomedical Sciences of Cells & Systems, University Medical Center Groningen, University of Groningen, Groningen, The Netherlands; 2 Department of Pathology & Medical Biology, University Medical Center Groningen, University of Groningen, Groningen, The Netherlands; 3 Department of Psychiatry, University Medical Center Groningen, University of Groningen, Groningen, The Netherlands

**Keywords:** Bipolar disorder, gastrointestinal permeability, gut-microbiome, major depressive disorder, probiotics, schizophrenia-spectrum disorders

## Abstract

Major depressive disorder (MDD), bipolar disorder (BD) and schizophrenia-spectrum disorders (SSD) are heterogeneous psychiatric disorders, which place significant burden on patient's well-being and global health. Disruptions in the gut-microbiome may play a role in these psychiatric disorders. This review presents current data on composition of the human gastrointestinal microbiota, and its interaction mechanisms in the gut–brain axis in MDD, BD and SSD. Diversity metrics and microbial relative abundance differed across studies. More studies reported inconsistent findings (*n* = 7) or no differences (*n* = 8) than studies who reported lower *α*-diversity in these psychiatric disorders (*n* = 5). The most consistent findings across studies were higher relative abundances of the genera *Streptococcus*, *Lactobacillus*, and *Eggerthella* and lower relative abundance of the butyrate producing *Faecalibacterium* in patients with psychiatric disorders. All three increased genera were associated with higher symptom severity. Confounders, such as medication use and life style have not been accounted for. So far, the results of probiotics trials have been inconsistent. Most traditional and widely used probiotics (consisting of *Bifidobacterium* spp. and *Lactobacillus* spp.) are safe, however, they do not correct potential microbiota disbalances in these disorders. Findings on prebiotics and faecal microbiota transplantation (FMT) are too limited to draw definitive conclusions. Disease-specific pro/prebiotic treatment or even FMT could be auspicious interventions for prevention and therapy for psychiatric disorders and should be investigated in future trials.

## Introduction

The human gut microbiome (GM), comprised of trillions of microorganisms, bacteria, viruses, fungi and other life forms, has been implicated in numerous aspects of human health and disease (Lai, Gao, & Zhang, 2020). The GM is diverse, personalized, dynamic and can be influenced, especially early in life, by factors such as vaginal/caesarean birth, diet, sleep, contact with other humans and stress (Gacesa et al., 2022; Szeligowski, Yun, Lennox, & Burnet, 2020). However, genetic factors, especially immunological background also determine a person's microbiome composition (Thaiss, Zmora, Levy, & Elinav, 2016). The GM partakes in a bidirectional communication with the central nervous system (CNS), called the gut–brain axis (Cryan et al., 2019). Disruptions to the GM have been associated with several neuropsychiatric disorders, including major depressive disorder (MDD), bipolar disorder (BD) and schizophrenia-spectrum disorders (e.g. schizophrenia, schizo-affective disorder and schizophreniform disorder, SSD) (Bastiaanssen et al., 2020; Genedi, Janmaat, Haarman, & Sommer, 2019; Nguyen, Hathaway, Kosciolek, Knight, & Jeste, 2021; Szeligowski et al., 2020).

MDD, BD and SSD are heterogeneous psychiatric disorders, which place significant burden on a patient's well-being and global health (WHO, 2006). Worldwide, MDD, BD and SSD affect 264 million, 45 million and 20 million people, respectively (James et al., 2018). Besides the overlap in psychiatric symptomatology, cognitive and biological functions, there is also a large genetic overlap between MDD, BD and SSD (Anttila et al., 2018). Nowadays it is generally assumed that SSD, BD and MDD are disorders that are not entirely separated, but represent different stages of a continuum of clinical pictures (Haarman, Riemersma-Van der Lek, Burger, Drexhage, & Nolen, 2016).

Research into the role of the GM in MDD, BD and SSD is still in its infancy. This review is a narrative review which we have approached in a structured way and it presents data on the development and composition of the human gastrointestinal microbiota, and its interaction mechanisms in the gut–brain axis in MDD, BD and SSD. Furthermore, the therapeutic potential of pre/probiotics, diet and faecal microbiota transplantation (FMT) in these disorders will also be discussed. We begin by showing the different routes between the gut and brain and will then discuss the microbiome, after that we will discuss gut permeability and the GM in the psychiatric disorders. Finally, we will discuss potential mechanisms on how to adapt the GM to improve the clinical situation of patients.

## Gut–brain axis

**Fig. 1.**
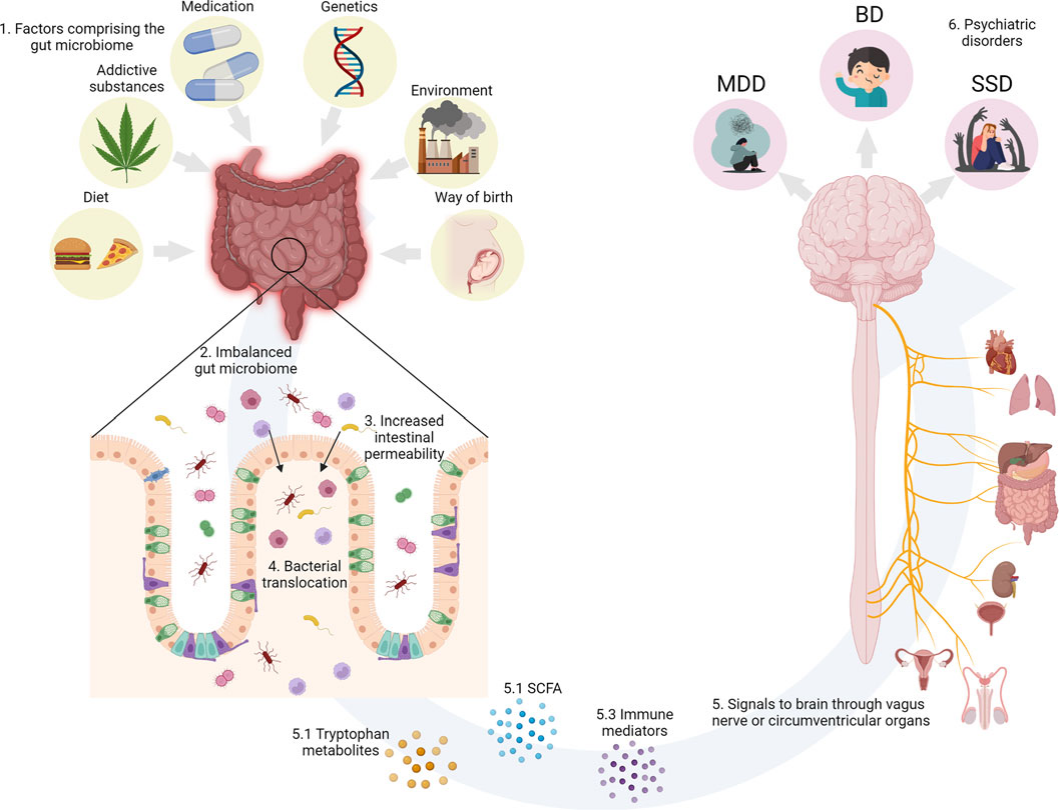
(1) Environmental factors known to impinge on the human GM. (2) GM dysbiosis impairs intestinal permeability. (3) Increased intestinal permeability causes translocation of luminal components and reactivity of the intestinal immune system. (4) Bacterial translocation activates the gut–brain axis. (5) The gut and the brain communicate bidirectionally via several routes, including the vagal nerve, the HPA axis, immune mediators such as cytokines, and the production of bacterial metabolites, such as SCFAs. (6) The environmental factors, GM dysbiosis and increased permeability separately and in concert could contribute the development of psychiatric disorders. Created with BioRender.com.

The gut and the brain communicate bidirectionally via several routes, including the vagal nerve, hypothalamic–pituitary–adrenal (HPA) axis, the production of bacterial metabolites, such as short-chain fatty acids (SCFAs), immune mediators and entero-endocrine signalling (Cryan et al., 2019; Golofast & Vales, 2020) (Fig. 1).

### Vagal nerve

The most direct route between the gut and the brain is the vagal nerve, roughly translated as the wandering nerve, and also known as the 10th cranial nerve. In reports from 1953 and 1961, ablation of the vagal nerve, formerly used for the treatment of peptic ulcers, resulted in an increase in the incidence of psychiatric disorders (Browning & Houseworth, 1953; Whitlock, 1961). Interestingly, this procedure recently turned out to reduce the incidence of Parkinson's disease (Svensson et al., 2015). In animals, Sgritta et al. (2019) observed that the effects of *Lactobacillus reuteri* on social behaviour were dissipated after vagotomy in a genetic mouse model of autism (*Shank3B^−/−^* mouse).

### Short-chain fatty acids

SCFAs are capable of signalling to the brain indirectly via nerve activation and can therefore influence behaviour (Sherwin, Sandhu, Dinan, & Cryan, 2016). Ninety five per cent of SCFAs consist of acetate, propionate and butyrate. The primary source of SCFAs is microbial fermentation of dietary fibres in the gut, which is supported by the fact that germ-free animals and antibiotic treatment results in low SCFA levels (Cryan et al., 2019). SCFAs are capable of modulating neurotransmission. For example, propionic acid increases tryptophan hydroxylase expression which can reduce indoleamine serotonin, thereby influencing serotonergic neurotransmission (El-Ansary, Bacha, & Kotb, 2012; Nankova, Agarwal, MacFabe, & La Gamma, 2014). So far, only a few studies have investigated relations between neuropsychiatric disorders and SCFAs. Skonieczna-żydecka et al. (2018) reported lower median content of acetate and higher isocaproic acid concentration in depressed women as compared to healthy women. Negative correlations were yielded between acetic, propionic and isocaproic acids and severity of depression as assessed with the Beck's Depression Inventory (BDI) scores. Szczesniak, Hestad, Hanssen, and Rudi (2016) reported significantly higher isovaleric levels in the depressed patients as compared to healthy subjects. However, Kelly et al. (2016) found no differences in acetate, propionate, isobutyrate and butyrate between depressed patients and healthy controls. The limited sample sizes in all three studies could explain these inconsistent results. In animal models of mania, sodium butyrate reversed mania-like, e.g. behavioural hyperactivity, and depressive-like behaviour (Moretti et al., 2011; Resende et al., 2013; Valvassori et al., 2015)

### Immune mediators

Immune mediators are important intermediaries between the gut microbiota and the brain. Cytokines can signal to the brain from the periphery via the vagal nerve or via the circumventricular organs (Sherwin et al., 2016). In the blood, bacteria or fragments from bacteria can bind lipopolysaccharide (LPS) binding protein (LBP), which is then connected to TLR-4, expressed on monocytes, macrophages and microglia, by soluble cluster of differentiation 14 (sCD14) (Kiecolt-Glaser et al., 2018; Kitchens & Thompson, 2005; Lim, Chang, & Wu, 2019), which can lead to nuclear factor kappa-light-chain enhancer of activated B cells (NF*κ*B) activation, inducing pro-inflammatory cytokine production (Genedi et al., 2019; Sherwin et al., 2016). Thus, the presence of bacteria or small parts of bacteria can activate the immune system which also affects the brain. Blood levels of LBP, sCD14 and NF*κ*B can reflect activity of this route. The group of Robert Yolken showed that these levels are increased in patients with BD and SSD, when patients had comorbid gastro-intestinal complaints (Severance et al., 2013; Severance, Dickerson, & Yolken, 2020). Moreover, Foster, Baker, and Dursun (2021) recently reviewed the relation of the GM and the immune system in MDD, where they draw attention to the importance of the immune system as an important player in the neurobiology combined with the GM in subtypes of depression.

### Tryptophan metabolites

Along with influencing other metabolites, gut-bacteria influence tryptophan metabolism (Carlessi, Borba, Zugno, Quevedo, & Réus, 2021). Tryptophan is an essential amino acid, which is metabolized by two main pathways, namely, the serotonin (5-HT) pathway and the kynurenine pathway. According to a meta-analysis of 101 studies tryptophan and kynurenine are decreased across MDD, BD and SSD (Marx et al., 2020). Conventional antidepressants enhance levels of central serotonin to produce a therapeutic effect. Most of the tryptophan is metabolized into kynurenine in the liver by tryptophan-2,3-dioxygenase for energy production, or following an inflammatory stimulus by indoleamine-2,3-dioxygenase. The presence of gut bacteria such as *Clostridium perfringens* can modulate gut production of 5-HT (Beaver & Wostmann, 1962; Yano et al., 2015). Studies in rats have shown that probiotic treatment can influence tryptophan, kynurenine and serotonin levels (Sherwin et al., 2016). Rats treated with *Bifidobacterium infantis 35624* had increased circulating tryptophan levels (Desbonnet, Garrett, Clarke, Bienenstock, & Dinan, 2009). Furthermore, rats treated with *Lactobacillus johnsonii* had lower circulating kynurenine levels with increased serotonin levels in the ileum and in the circulation (Valladares et al., 2013). Moreover, one study in MDD patients found decreases in the kynurenine/tryptophan ratio after treatment with probiotics (*Lactobacillus helveticus* and *Bifidobacterium longum*) (Kazemi, Noorbala, Azam, Eskandari, & Djafarian, 2019). A meta-analysis studying the effect of probiotic supplementation on the tryptophan–kynurenine pathway observed significantly lower serum kynurenine and a decreased kynurenine/tryptophan ratio after treatment (Purton et al., 2021). The results so far provide preliminary evidence that probiotics can modulate the tryptophan–kynurenine pathway.

## Gut microbiome

The GM contains 10^14^ microorganisms, 2–20 million unique genes and over 1000 unique bacterial species. The GM is a perplexing genomic structure with many more genes than the human genome (Golofast & Vales, 2020). In contrast to the human genome, which is unalterable over lifetime, the GM is highly adaptable (Nguyen et al., 2021). The GM is already influenced by the environment at the day of birth. Vaginally delivered neonates' microbiota resembles the maternal vaginal and faecal bacteria, whereas for infants born by caesarean section, their microbiota resembles the maternal skin and hospital environment (Bäckhed et al., 2015; Korpela et al., 2020; Mitchell et al., 2020). In 2002 Cannon, Jones and Murray already identified unplanned caesarean section as a risk factor for schizophrenia in their seminal meta-analysis. One epidemiological study found a weak association between birth by planned caesarean section, but not by unplanned emergency caesarean section and the risk of developing psychosis or BD (O'Neill et al., 2016). However, after correcting for matched siblings, this effect was no longer significant. The Finnish birth register data also showed an odds ratio (OR) of 2.5 for BD for birth by caesarean section (Chudal et al., 2014).

Associations between antibiotic exposure and psychiatric disorders have been found in multiple large population-based studies (Köhler et al., 2017; Liang et al., 2021; Lurie, Yang, Hayne, Mamtani, & Boursi, 2015). Lurie et al. (2015) found that treatment with a single antibiotic course was associated with higher risk for depression (*n* = 202,974) compared to a healthy control group (*n* = 803,961), with adjusted ORs of 1.23 [95% confidence level (CL) 1.18–1.29] for penicillin's and 1.25 (95% CL 1.15–1.35) for quinolones. Köhler et al. (2017) performed a large-scale study in individuals born in Denmark in 1985–2002 (*n* = 1,015,447), of which 5759 individuals were diagnosed with schizophrenia. The association of infections treated with anti-infective agents and the subsequent risk of schizophrenia and affective disorders during 1995–2013 was studied. Antibiotics, with a hazard rate ratio (HRR) of 1.44 (95% CL 1.25–1.66), and especially broad spectrum antibiotics (HRR = 1.53; 95% CL 1.32–1.71) were associated with increased risk for schizophrenia. However, Lurie et al. (2015) did not find an association between antibiotic use and psychosis (*n* = 8487). Liang et al. (2021) observed positive associations between long-term antibiotic use during early life (defined by the UK Biobank as child or teenager) and anxiety and depression. Lastly, there are also studies showing that antibiotic use can induce hypomania or mania, also known as antibiomania (Abouesh, Stone, & Hobbs, 2002). In a review of 47 published cases, clarithromycin, a broad spectrum antibiotic, was related to (hypo)mania in 16 out of the 47 cases (Lambrichts, Van Oudenhove, & Sienaert, 2017). One explanation could be that the administration of antibiotics can result in changes in the microbiome which could in turn increases the risk of (hypo)mania, for example by lowering the bacterial production of gamma-amino-butyric acid (Dickerson, Severance, & Yolken, 2017).

## Gastrointestinal permeability in patients with severe psychiatric disorders

### Gastrointestinal barrier

The gastrointestinal barrier is a dynamic, multilayer system which consists of a physical barrier and a biochemical barrier. The main components of the physical barrier are the epithelial cells sealed by tight junctions and the gut mucosa. The biochemical barrier consists of the gut microbiota and the mucosal immune system. Its main function is to keep pathogens out of the host's internal milieu, while at the same time facilitate the absorption of nutrients, water and electrolytes (Bischoff et al., 2014). For the homoeostasis of the organism, the permeability of the gastrointestinal barrier needs to be maintained within a narrow equilibrium.

Diet and lifestyle factors such as energy-dense food and alcohol can disturb gut permeability and lead to translocation of luminal components and reactivity of the intestinal immune system (Bischoff et al., 2014). Increased intestinal permeability can also be a result of changes in gut microbiota, infections or reduced perfusion of the gut (Bischoff et al., 2014).

### Gastrointestinal permeability and brain structure and function

Gastrointestinal permeability and brain structure and function are governed by a bidirectional interaction. On one hand, pre-clinical studies (Keita, Söderholm, & Ericson, 2010; Kiliaan et al., 1998; Söderholm et al., 2002; Vicario et al., 2010) and studies in healthy volunteers (Vanuytsel et al., 2014) suggest a causal effect of psychosocial stress on gut permeability, likely through corticotropin-releasing hormone-mediated mast cell activation and decreased blood flow to the gut during stressful periods. On the other hand, it is hypothesized that increased gut permeability and abnormal influx of food- and bacteria-derived antigens drives systemic low-grade immune dysregulation, which in turn affects brain structure and function (Genedi et al., 2019). Lastly, psychiatric comorbidity is common in diseases with known structural and functional abnormalities of the gastrointestinal barrier, such as Crohn's disease and colitis ulcerosa (Bennett, Tennant, Piesse, Badcock, & Kellow, 1998; Faresjö et al., 2007; Nicholl et al., 2008). It is therefore hypothesized that abnormal intestinal permeability is involved both as an effect and as a cause of severe psychiatric disorder, yet, this is still a research area in its infancy.

### Evidence of gastrointestinal barrier dysfunction in MDD, BD and SSD

In MDD, BD and SSD, gastrointestinal permeability has been assessed with markers of structural barrier integrity and paracellular permeability such as zonulin and intestinal-type fatty acid-binding protein (I-FABP) (Table 1). In a study in MDD or anxiety disorder, authors reported significantly higher levels of zonulin and I-FABP in patients compared to controls and this was associated with gut dysbiosis (Stevens et al., 2018). Alvarez-Mon et al. (2019) also reported significantly higher levels of I-FABP in MDD patients compared to controls but no significant difference was observed for zonulin. In another study, patients of different psychiatric diagnoses who had recently attempted suicide had higher levels of I-FABP but lower levels of zonulin compared to both MDD patients without a history of suicide attempt and healthy controls (Ohlsson et al., 2019). In BD, patients in full remission showed higher zonulin and claudin-5 compared to controls, while there were no differences between active manic episode and remission (Kılıç, Işık, Demirdaş, Doğuç, & Bozkurt, 2020). Interestingly, this observation might suggest that zonulin and claudin-5 are ‘trait’ rather than ‘state’ markers of BD. However, the small sample size of this study precludes firm conclusions, before the findings are confirmed in larger populations. Moreover, there is a certain level of uncertainty surrounding the measurement of zonulin with commercially available ELISA kits. The results should be interpreted with caution until an in depth understanding of the biomarker is acquired (Massier, Chakaroun, Kovacs, & Heiker, 2021). In schizophrenia, Maes, Kanchanatawan, Sirivichayakul, and Carvalho (2019a) and Maes, Sirivichayakul, Kanchanatawan, and Vodjani (2019b) showed that immunoglobulin M (IgM) responses to zonulin were higher in patients compared to controls, while IgM response to occludin was significantly associated with deficit schizophrenia. Authors also reported an increased ratio of IgM towards components of the paracellular route (zonulin + occludin)/components of the transcellular route (talin + actin + vinculin) in deficit *v.* non-deficit schizophrenia and in schizophrenia *v.* controls. This ratio was significantly associated with increased IgA responses to Gram-negative bacteria. Schizophrenia patients with deficit syndrome (i.e. severe negative and cognitive symptoms) can be expected to have unhealthy dietary habits, which could explain the association with IgM response to occludin. Barber et al. (2019) showed that 42.9% of the patients had higher levels of zonulin than the cut-off for elevated levels (>2.33 mg/dl).

**Table 1.** Main findings of studies assessing gut permeability in MDD, BD and SSD

Measure of gastrointestinal permeability	Reference	Diagnosis	Sample size	Main findings
*Markers of paracellular and transcellular disruption*				
I-FABP Zonulin LPS	Stevens et al. (2018)	MDD or anxiety disorder	MDD or anxiety disorder: *n* = 22 HC: *n* = 28	↑plasma levels of LPS, zonulin and I-FABP in patients compared to controls Gut dysbiosis in patients, not in controls
LBP I-FABP Zonulin	Alvarez-Mon et al. (2019)	MDD	MDD: *n* = 22 HC: *n* = 14	↑levels of circulating LBP, and I-FABP in patients compared to controls No significant differences in levels of zonulin
I-FABP Zonulin sCD14	Ohlsson et al. (2019)	Different psychiatric diagnosis with recent suicide attempt (rSA) and MDD without history of suicide attempt (nsMDD)	rSA: *n* = 54 nsMDD: *n* = 13 HC: *n* = 17	↑I-FABP and ↓zonulin levels in the group with recent suicide attempters compared to both the MDD group without history of suicide attempt and the healthy controls
Zonulin Claudin-5	Kılıç et al. (2020)	BD	BD: *n* = 41 HC: *n* = 41	↑zonulin and claudin-5 levels in patients compared to healthy controls. No difference in zonulin and claudın-5 levels between patients with manic episodes and patients in remission
IgM against zonulin, occluding, talin, actin and vinculin IgA against Gram-(−) bacteria	Maes, Sirivichayakul, Kanchanatawan, and Vodjani (2019b)	SSD	SSD: *n* = 78 HC: *n* = 40	↑ratio of IgM to zonulin + occludin/talin + actin + vinculin (PARA/TRANS) in deficit *v.* non-deficit schizophrenia and in schizophrenia *v.* controls PARA/TRANS ratio significantly associated with increased IgA responses to Gram-(−) bacteria IgM to zonulin positively associated with schizophrenia (*v.* controls)
Zonulin	Barber et al. (2019)	SSD	SSD: *n* = 98	42.9% of the patients had higher levels of zonulin than the cut-off for elevated levels (>2.33 mg/dl)
*Markers of bacterial translocation*				
sCD14 LBP	Severance et al. (2013)	BD SSD	BD: *n* = 75 SSD: *n* = 141 HC: *n* = 78	↑sCD14 in BD and SSD compared to HC ↑LBP in SCZ compared to BD
IgM/IgA against LPS of Gram-(−) bacteria	Maes et al. (2013)	MDD	MDD: *n* = 113 HC: *n* = 28	↑IgM/IgA against the LPS of Gram-(−) bacteria in MDD compared to controls
sCD14	Tanaka et al. (2017)	BD SCZ HC	BD: *n* = 32 SCZ: *n* = 28 HC: *n* = 60	↑sCD14 in BD and SCZ compared to HC
sCD14	Morch et al. (2019)	SSD HC	SSD: *n* = 675 HC: *n* = 647	sCD14 was not significantly different between SSD and HC
IgM/IgA against LPS of Gram-(−) bacteria	Simeonova et al. (2020)	MDD BD	MDD: *n* = 44 BD: *n* = 66	IgA/IgM against LPS of gut commensal bacteria were positively associated with MDD and BD as compared to healthy controls
IgM/IgA against Gram-(−) bacteria	Maes et al. (2019a, 2019b)	SSD	SSD: *n* = 80 HCL: *n* = 38	IgM/IgA against gut commensal bacteria were associated with negative symptoms, neurocognitive impairments and the deficit phenotype of SSD
sCD14	Dzikowski et al. (2020)	SCZ HC	SCZ: *n* = 50 HC: *n* = 60	↑sCD14 in SCZ compared to HC

sCD14, soluble cluster of differentiation 14; LBP, lipopolysaccharide (LPS) binding protein; I-FABP, fatty acid-binding protein.

Markers of bacteria translocation can also reflect abnormal gastrointestinal permeability. LPS and LBP were up-regulated in MDD or anxiety disorder (Alvarez-Mon et al., 2019; Stevens et al., 2018). In BD, sCD14, a co-receptor for the LBP, used as a marker for bacterial translocation was significantly higher in patients compared to controls (Severance et al., 2013). In SSD, sCD14 was significantly higher in multiple studies (Dzikowski et al., 2020; Severance et al., 2013; Tanaka et al., 2017; Weber et al., 2018) but not all (Morch et al., 2019). In regard to antibodies against gut commensal bacteria, in MDD, Maes et al. (2013) showed increased IgM/IgA against Gram-negative bacteria. More recently, Simeonova et al. (2020) showed increased immune responses to Gram-negative bacteria in both MDD and BD, especially in the presence of melancholia, compared to healthy controls. Interestingly, IgA/IgM response profiles differ among the two diagnosis, as well as between BD I and II subtypes and patients with melancholia. This might reflect a common underlying disruption of the gut barrier across diagnoses, accompanied however by distinct microbiome profiles and immune susceptibilities, though this remains to be confirmed in future studies. In SSD IgM/IgA against gut commensal bacteria were associated with negative symptoms, neurocognitive impairments and the deficit phenotype of SSD (Maes et al., 2019b).

## GM in three major psychiatric disorders

### Alpha and beta diversity

*α*-Diversity is the mean species diversity in sites or habitats at a local scale (Whittaker, 1972). *α*-Diversity is often measured by the Fisher, Ace, Chao, Simpson and/or Shannon indices. *β*-Diversity shows differentiation among habitats (Whittaker, 1972). Multiple studies analysed diversity metrics; the findings however were mixed.

For MDD studies (Table 2), *α*-diversity was not different from controls in three studies (Chung et al., 2019; Zheng et al., 2016, 2020). Two studies found lower *α*-diversity in MDD (Huang et al., 2018; Liu et al., 2016). Four studies found inconsistent findings across indices (Jiang et al., 2015; Kelly et al., 2016; Lai et al., 2021; Rong et al., 2019). *β*-Diversity was significantly different between MDD and controls in four studies (Huang et al., 2018; Kelly et al., 2016; Lai et al., 2021; Zheng et al., 2016) and not different in three studies (Chung et al., 2019; Jiang et al., 2015; Rong et al., 2019).

**Table 2.** Studies of the GM in major depressive disorder

Study design	Alpha and beta diversity	Significantly more abundant taxa in the MDD group	Significantly more abundant taxa in the control group	Association with clinical features
^A^**Study group**: HC (*n* = 63); MDD (*n* = 58) **Mean age (s.d.)**: HC: 41.8 (12.3); MDD: 40.6 (11.7) **Sex (F/M):** HC: 40/23; MDD: 36/22 **HDRS Scores (s.d.)**: HC: 0.3 (0.7); MDD: 22 (2.4) **Experimental method**: 16S rRNA gene sequencing from faecal samples	*α*: No differences (observed species, Shannon index, phylogenetic diversity and Simpson) *β*: Different (unweighted and weighted UniFrac analysis)	**Phyla**: *Actinobacteria*	**Phyla**: *Bacteroidetes*	
^B^**Study group**: HC (*n* = 30); MDD (*n* = 29) **Mean age (s.d.)**: HC: 26.8 (5.4); MDD: 25.3 (5.4) **Sex (F/M):** HC: 15/15; MDD: 11/18 **MADRS scores (s.d.)**: MDD: 27.4 (8.5) **HAMDS scores (s.d.)**: MDD: 29.8 (7.6) **Experimental method**: 16S rRNA gene 454 sequencing (Roche) from faecal samples	*α*: One index was significantly higher in the MDD group (Shannon index), three other measurements were not different (Ace, Chao 1 and Simpson index) *β*: Not different (UniFrac analysis)	**Phylum**: *Fusobacteria*, *Proteobacteria*, *Bacteroidetes* **Family**: *Acidaminococcaceae*, *Enterobacteriaceae*, *Fusobacteriaceae*, *Porphyromonadaceae*, *Rikenellaceae* **Genus**: *Alistipes*, *Blautia*, *Clostridium XIX*, *Lachnospiraceae incertae sedis*, *Megamonas*, *Parabacteroides*, *Parasutterella*, *Phascolarctobacterium*, *Oscillibacter*, *Roseburia*	**Phylum**: *Actinobacteria*, *Firmicutes* **Family**: *Bacteroidaceae*, *Erysipelotrichaceae*, *Lachnospiraceae*, *Prevotellaceae*, *Ruminococcaceae*, *Veillonellaceae* **Genus**: *Bacteroides*, *Dialister*, *Faecalibacterium*, *Prevotella*, *Ruminococcus*	Negative relationships between the relative abundance of *Faecalibacterium* and depressive symptom severity in MADRS and HAMDS
^C^**Study group**: HC (*n* = 33); MDD (*n* = 34) **Mean age (s.d.)**: HC: 45.8 (11.9); MDD: 45.8 (11.5) **Sex (F/M):** HC: 14/19; MDD: 13/21 **Beck Depression (s.d.)**: MDD: 32.4 (9.92) **HAMD 17 median (range)**: MDD: 19.5 (14) **Experimental method**: 16S rRNA gene sequencing (Illumina MiSeq platform) from faecal samples	*α*: Chao 1, total observed species and phylogenetic diversity were decreased in the MDD group. Shannon index was not significantly different. Richness: *β*: Different (Bray–Curtis, unweighted and weighted UniFrac)	**Family**: *Thermoanaerobacteriaceae* **Genus:** *Eggerthella*, *Holdemania*, *Gelria*, *Turicibacter*, *Paraprevotella*, *Anaefilum*	**Family**: *Prevotellaceae* **Genus**: *Prevotella*, *Dialister*	
^D^**Study group**: HC (*n* = 37); MDD (*n* = 36) **Mean age (s.d.)**: HC: 41.19 (12.73); MDD: 45.83 (14.08) **Sex (F/M):** HC: 23/14; MDD: 28/8 **Beck Depression (s.d.)**: HC: 4.54 (4.85), MDD: 19.18 (12.47) **Experimental method**: 16S rRNA gene sequencing (Illumina MiSeq platform) from faecal samples	*α*: No differences (Shannon index) *β*: No differences (weighted and unweighted UniFrac)	**Phylum**: *Firmicutes*, *Actinobacteria* **Family**: *Peptostreptococcaceae*, *Porphyromonadaceae*, *Streptococcaceae*, *Bifidobacteriaceae*, *Lachnospiraceae* **Genus**: *Clostridium XI*, *Holdemania*, *Adlercreutzia*, *Eggerthella*, *Parabacteroides*, *Streptococcus*, *Ruminococcus*, *Bifidobacterium*, *Blautia*	**Phylum**: *Proteobacteria*, *Bacteroidetes* **Family**: *Alcaligenaceae*, *Prevotellaceae* **Genus**: *Megamonas*, *Sutterella*, *Prevotella*	Families *Peptostreptococcaceae* and Porphyromonadaceae show positive correlations with BDI scores. Families *Prevotellaceae* and *Alcaligenaceae* show negative correlations with BDI scores. At the genus level *Blautia*, *Ruminococcus*, *Parabacteroides*, *Eggerthella* and *Clostridium XI* show positive correlations with BDI scores. *Prevotella* and *Sutterella* show negative correlations with BDI scores.
^E^**Study group**: HC (*n* = 27); MDD (*n* = 27) **Mean age (s.d.)**: HC: 42.3 (14.1); MDD: 48.7 (12.8) **Sex (F/M):** HC: 20/7; MDD: 20/7 **Experimental method**: 16S rRNA gene sequencing (Illumina HiSeq2500) from faecal samples	*α*: Significantly lower in the MDD group (ACE, Chao 1, Shannon and Faith's phylogenetic diversity) *β*: Different (weighted and unweighted UniFrac)	**Genus**: *Oxalobacter*, *Parvimonas*, *Bulleidia*, *Pseudomonas*, *Peptostreptococcus*, *Gemella*	**Phylum**: *Firmicutes* **Family:** *Lachnospiraceae*, *Ruminococcaceae*, *Clostridiaceae*, **Genus**: *Blautia*, *Faecalibacterium*, *Dorea*, *Coprococcus*	
^F^**Study group**: HC (*n* = 29); MDD (*n* = 26) **Mean age (s.d.)**: HC: 39.41 (10.96); MDD: 43.73 (11.46) **Sex (F/M):** HC: 16/13; MDD: 18/8 **HAMD (s.d.):** 19.81 (2.95) **Experimental method**: Shotgun metagenomics – Illumina HiSeq2500	*α*: Fischer index was significantly lower in the MDD group. Shannon index showed no differences between the groups. *β*: Different (PCoA plots, Bray–Curtis index)	**Phylum**: *Actinobacteria* **Family**: *Bifidobacteriaceae*, *Micrococcaceae*, *Atopobiaceae*, *Eggerthellaceae*, *Enterococcaceae*, *Oscillospiraceae*, *Peptococcaceae*, *Acidaminococcaceae*, *Veillonellaceae* **Genus**: *Slackia*, *Eggerthella*, *Coriobacterium*, *Lactobacillaceae*, *Olsenella*, *Atopobium*, *Rothia*, *Bifidobacterium*, *Enterococcus*, *Lactobacillus*, *Streptococcus*, *Heliobacterium*, *Lachnoclostridium*, *Oscillibacter*, *Desulfitobacterium*, *Acidaminococcus*, *Megaspheara*, *Sphaerotheata* **Species**: *Bifidobacterium adolescentis*, *Bifidobacterium longum*, *Bifidobacterium dentium*, *Bifidobacterium bifidum*, *Bifidobacterium breve m breve*	**Phylum**: *Bacteroidetes* **Family**: *Bacteroidaceae*, *Cytophagaceae*, *Flavobacteriaceae*, *Sphingobacteriaceae* **Genus**: *Bacteroides*, *Sphingobacterium*	
^G^**Study group**: HC (*n* = 20); MDD (*n* = 15) **Mean age (s.d.)**: HC: 43.9 (11.2); MDD: 44.8 (14.9) **Sex (F/M):** HC: 13/7; MDD: 11/4 **HAMD (s.d.):** 19.81 (2.95) **Experimental method**: 16S rRNA gene 454 sequencing (Roche) from faecal samples	*α*: Significantly lower in MDD group (Shannon index) *β*: Not reported	**Genus**: *Prevotella*, *Bacteroides*, *Paraprevotella*, *Dialister*, *Veillonella*, *Haemophilus*	**Genus**: *Bifidobacterium*, *Barnesiella*, *Odoribacter*, *Butyricimonas*, *Alistipes*, *Parabacteroides*, *Faecalibacterium*, *Lachnospiracea_incertae_sedis*, *Blautia*, *Coprococcus*, *Ruminococcus*, *Mituokella*, *Megamonas*, *Clostridium XI*, *Oscillibacter*, *Clostridium sensu stricto*, *Clostridium IV*, *Roseburia*, *Acetivibrio*, *Fusobacterium*, *Escherichia*/*Shigella*, *Gemmiger*, *Comamonas*, *Sutterella*, *Vampirovibrio*	
^H^**Study group**: HC (*n* = 171); MDD (*n* = 122) **Mean age (s.d.)**: HC: 26.85 (5.48); MDD: 26.54 (4.07) **Sex (F/M):** HC: 100/71; MDD: 77/45 **HAMD (s.d.)**: 22.65 (5.5) **Experimental method**: 16S rRNA gene sequencing from stool samples	*α*: No differences (ACE, Chao, inverse Simpson and Shannon) *β*: Significantly different with PC2 PLS-DA analysis, but not with PC1 PLS-DA analysis	**Phylum**: *Bacteroidetes* **Family**: *Bacteroidaceae*, *Bifidobacteriaceae*	**Family**: *Enterobacteriaceae*	*Peptostreptococcaceae* OTU901 was negatively correlated with the HAMD
^I^**Study group**: HC (*n* = 30); MDD (*n* = 31) **Mean age (s.d.)**: HC: 39.47 (10.22); MDD: 41.58 (10.40) **Sex (F/M):** HC: 22/9; MDD: 22/9 **HAMD (s.d.):** 20.23 (3.11) **HCL-32**: 6.68 (7.15) **Experimental method**: Shotgun metagenomics Illumina HiSeq2500	*α*: Shannon index and the inverse Simpson index were not significantly different between groups *G*_m_ coefficient and Chao 1 were significantly lower in the MDD group. *β*: No differences (Bray–Curtis index)	**Phylum**: *Firmicutes*, *Actinobacteria* **Genera**: *Clostridium*, *Bifidobacterium*, *Oscillibacter*, *Streptococcus*, *Selenomonas*, *Megasphaera*, *Acidaminococcus*, *Treponema*, *Lactobacillus*, *Ethanoligenens*, *Enterococcus*, *Cellulosilyticum*, *Eggerthella*, *Desulfovibrio*, *Desulfitobacterium*, *Sphaerochaeta*, *Desulfotomaculum*, *Heliobacterium* **Species**: *Eubacterium rectale ATCC 33656*, *Enterobacteriaceae*, *Prevotella*, *Clostridium saccharolyticum WM1*, *Escherichia coli*, *Megasphaera elsdenii DSM 20460*, *Oscillibacter valericigenes Sjm18-20*, *Bifidobacterium adolescentis ATCC 15703*, *Eubacterium rectale*, *Prevotella melaninogenica ATCC 25845*, *Prevotella intermedia 17*, *Prevotella denticola F0289*, *Bifidobacterium*, *Selenomonas ruminantium* subsp. *Lactilytica TAM6421*, *Akkermansia muciniphila ATCC BAA-835*, *Bifidobacterium longum*, *Selenomonas sputigena ATCC 35185*, *Acidaminococcus intestine RyC-MR95*, *Bifidobacterium dentium_Bd1*	**Phylum**: *Bacteroidetes* **Genera**: *Bacteroides*, *Odoribacter*, *Tannerella*, *Veillonella*, *Haemophilus*, *Porphyromonas*, *Paludibacter* **Species**: *Haemophilus parainfluenzae T3T1*	
^J^**Study group**: HC (*n* = 47); MDD (*n* = 43) **Mean age (s.d.)**: HC: 22.1 (1.8); MDD: 21.9 (2.1) **Sex (F/M):** HC: 34/13; MDD: 38/5 **PROMIS Depression Score (s.d.):** HC: 9.3 (1.4); MDD: 25 (6.9) **Experimental method**: 16S rRNA gene sequencing from stool samples	*α*: Faith's phylogenetic diversity was significantly lower in the MDD group. Shannon index was significantly different. *β*: Significantly different (Bray–Curtis index and UniFrac distance)	**Phylum**: *Bacteroidetes* **Class**: *Bacteroidia*, *Gammaproteobacteria* **Order**: *Bacteroidales* **Family**: *Enterococcaceae* **Genera**: *Flavonifractor*, *Sellimonas*, *Enterococcus*	**Phylum**: *Firmicutes* **Class**: *Clostridia* **Order**: *Clostridiales*, *Rhodospirillales* **Family**: *Ruminococcaceae*, *Christensenellaceae*, *Barnesiellaceae* **Genera**: *Faecalibacterium*, *Subdoligranulum*, *Coprostanoligenes* group, *Ruminococcus 1*, *Fusicatenibacter*, *Tyzzerella 3*, *Ventrosium* group, *Barnesiella*, *Desulfovibrio*	Phylum *Firmicutes*, class *Clostridia*, order *Clostridiales* and phyla *Ruminococcaceae*, *Faecalibacterium and Coprostanoligenes* group were more reduced in patients with more severe symptoms. Phylum *Bacteroidetes*, class B*acteroidia* and *Gammaproteobacterial*, order B*acteroidales* and genera *Flavinofractor* and *Sellimonas* were more increased in patients with more severe symptoms.

HC, healthy controls; MDD, major depressive disorder; PCoA, principal coordinates analysis; PLS-DA, partial least-squares discriminant analysis; HDRS, Hamilton Depression Rating Scale; MADRS, Montgomery–Åsberg Depression Rating Scale; HAMDS, Hamilton's Depression Scale; PROMIS Depression Score: Patient-Reported Outcomes Measurement Information System Depression Score; OTU, operational taxonomic unit.

^A^Zheng et al. (2016); ^B^Jiang et al. (2015); ^c^Kelly et al. (2016); ^D^Chung et al. (2019); ^E^Huang et al. (2018); ^F^Lai et al. (2021); ^G^Liu et al. (2016); ^H^Zheng et al. (2020); ^I^Rong et al. (2019); ^J^Liu et al. (2020).

For *α*-diversity in the BD studies (Table 3), three had inconsistent findings (Hu et al., 2019; Painold et al., 2018; Rong et al., 2019) and one found no differences (Zheng et al., 2020). *β*-Diversity was different in two studies (Hu et al., 2019; Zheng et al., 2020) and two found no differences (Painold et al., 2018; Rong et al., 2019).

**Table 3.** Studies of the GM in individuals with BD

Study design	Microbial richness/diversity	Significantly more abundant taxa in the BD group	Significantly more abundant taxa in the control group	Association with clinical features
^T^**Study group**: HC (*n* = 45); BD (*n* = 52) **Mean age (s.d.)**: HC: 36.29 (12.22); BD: 24.15 (9.5) **Sex (F/M):** HC: 22/23; BD: 25/27 **MADRS (s.d.)**: 28.15 (8.85) **HDRS-17 (s.d.**): 30.15 (8.31) **YMRS (s.d.)**: 1.87 (1.43) **Experimental method**: 16S rRNA gene sequencing from stool samples	*α*: Greater *α*-diversity in HC when measured with Obs, Chao 1 and incidence-based coverage estimators. However, no differences when measured with Shannon, Simpson or inverse Simpson indices. *β*: Different (PCoA)	**Phylum**: *Bacteroidetes* **Class**: *Flavobacteria*, *Bacteroidia* **Order**: *Oceanospirillales*, *Flavobacteriales*, *Bacteroidales* **Family**: *Halomonadaceae*, *Flavobacteriaceae*, *Porphyromonadaceae*, *Bacteroidaceae* **Genera**: *Weissella*, *Anaerofustis*, *Halomonas*, *Parabacteroides*, *Bacteroides*	**Phylum**: *Firmicutes* **Class**: *Clostridia* **Order**: *Clostridiales*, *Rhizobiales* **Family**: *Lachnospiraceae*, *Hyphomicrobiaceae* **Genera**: *Roseburia*, *Faecalibacterium*, *Ruminococcus*, *Gemmiger*, *Parasutterella*, *Coprococcus*	MADRS scores were negatively correlated with *Acetanaerobacterium*, *Stenotrophomonas*, *Anaerotruncus* and *Raoultella*, but positively correlated with *Acinetobacter* and *Cronobacter*
^U^**Study group**: HC (*n* = 171); BD (*n* = 169) **Mean age (s.d.)**: HC: 26.85 (5.48); BD: 25.59 (8.41) **Sex (F/M):** HC: 100/71; BD: 84/85 **HAMD (s.d.)**: 26.13 (9.79) **YMRS (s.d.)**: 3.24 (4.43) **Experimental method**: 16S rRNA gene sequencing from stool samples	*α*: Greater *α*-diversity in HC when measured with Ace and Chao 1. However, no differences when measured with inverse Simpson and Shannon indices *β*: Different (PLS-DA, PC1 PLS-DA, PC2 PLS-DA)	**Phylum**: *Proteobacteria*, *Fusobacteria*, *Saccharibacteria*, *Synergistetes* **Family**: *Pseudomonadaceae*	**Phylum**: *Bacteroidetes*	Not reported
^V^**Study group**: HC (*n* = 10); BD (*n* = 32) **Mean age (s.d.)**: HC: 31.4 (7.61); BD: 41.31 (17.73) **Sex (F/M):** HC: 6/4; MDD: 14/18 **HAMD (s.d.)**: 6.94 (4.37) **BDI (s.d.)**: 16.45 (11.41) **Experimental method**: 16S rRNA gene sequencing from stool samples	*α*: No differences (observed species, Chao 1, Shannon and Simpson indices) *β*: No differences (weighted PCoA UniFrac and unweighted PCoA UniFrac)	**Phylum**: *Actinobacteria* **Class**: *Coriobacteria* **Family**: *Coriobacteriaceae* **Order**: *Coriobacteriaceae*	**Family**: *Ruminococcaceae* **Order**: *Faecalibacterium*	No significant association of microbial diversity with depression levels was found
^W^**Study group**: HC (*n* = 30); BD (*n* = 30) **Mean age (s.d.)**: HC: 39.47 (10.22); BD: 38.4 (8.33) **Sex (F/M):** HC: 22/9; MDD: 15/15 **HAMD (s.d.):** 20.37 (3.41) **HCL-32**: 20.23 (4.58) **Experimental method**: Shotgun metagenomics Illumina HiSeq2500	*α*: *G*_m_ coefficient was significantly lower in the BD group. Chao, Shannon and inverse Simpson indices did not differ. *β*: No differences (Bray–Curtis)	**Phylum:** *Firmicutes*, *Proteobacteria*, *Actinobacteria* **Genera**: *Escherichia*, *Clostridium*, *Bifidobacterium*, *Oscillibacter*, *Klebsiella*, *Streptococcus*, *Selenomonas*, *Megasphaera*, *Acidaminococcus*, *Veillonella*, *Treponema*, *Lactobacillus*, *Ethanoligenens*, *Enterococcus*, *Cellulosilyticum*, *Eggerthella*, *Enterobacter*, *Desulfovibrio*, *Shigella*, *Desulfitobacterium*, *Sphaerochaeta*, *Salmonella*, *Desulfotomaculum*, *Heliobacterium* **Species**: *Enterobacteriaceae*, *Eubacterium rectale ATCC 33656*, *clostridium saccharolyticum WM1*, *Escherichia coli*, *Oscillibacter valericigenes Sjm18-20*, *Bifidobacterium adolescentis ATCC 15703*, *Akkermansia muciniphila ATCC BAA-835*, *Megasphaera elsdenii DSM 20460*, *Klebsiella*, *Bifidobacterium*, *Selenomonas ruminantium* subsp. *Lactilytica TAM6421*, *Ethanoligenens harbinense YUAN-3*, *Acidaminococcus fermentans DSM 20731*, *Acaidaminococcus intensi RyC MR95*, *Selenomonas sputigena ATCC 35185*	**Phylum**: *Bacteroidetes* **Genera**: *Bacteroides*, *Odoribacter*, *Tannerella*, *Haemophilus*, *Porphyromonas*, *Paludibacter* **Species**: *Bacteroides helcogenes p 36–108*, *Bacteroidetes helcogenes*, *Bacteroidetes*, *Haemophilus parainfluenzae T3T1*, *Klebsiella pneumoniae*	Not reported

HC, healthy controls; BD, bipolar disorder; PLS-DA, partial least-squares discriminant analysis; PCoA, principal coordinates analysis; HCL-32, Hypomania Check List-32; HAMD, Hamilton's Depression Scale; BDI, Beck Depression Inventory; MADRS, Montgomery–Åsberg Depression Rating Scale; HDRS-17, 17-item Hamilton Depression Rating Scale.

^T^Hu et al. (2019); ^U^Zheng et al. (2020); ^V^Painold et al. (2018); ^W^Rong et al. (2019).

In SSD (Table 4), three studies reported lower *α*-diversity in the SSD groups compared to healthy controls (Ma et al., 2020; Xu et al., 2020; Zheng et al., 2019), four reported no differences (He et al., 2018; Li et al., 2020; Nguyen et al., 2018; Shen et al., 2018), two did not report on *α*-diversity (Schwarz et al., 2018; Yuan et al., 2018) and one reported higher *α*-diversity in the SSD group (Zhu et al., 2020b). *β*-Diversity was different between groups in five studies (He et al., 2018; Nguyen et al., 2021; Shen et al., 2018; Xu et al., 2020; Zheng et al., 2019), from those five, two studies reported tighter clustering in the healthy control group (Nguyen et al., 2018; Shen et al., 2018).

**Table 4.** Studies of the GM in schizophrenia-spectrum disorder

Study design	Alpha and beta diversity	Significantly more abundant taxa in the SSD group	Significantly more abundant taxa in the control group	Association with clinical features
^K^**Study group**: Schizophrenia patients (*n* = 63) and healthy controls (*n* = 69) **Mean age (s.d.)**: HC: 39.99 (1.62); SSD: 43.49 (1.68) **Sex (F/M)**: HC: 33/36; SSD: 21/42 **PANSS (s.d.)**: 71.87 (1.85) **Experimental method**: 16S rRNA gene sequencing from faecal samples	*α*: Shannon and Chao indices were significantly lower in the SSD group *β*: Different at the OTU level (PLS-DA)	**Families**: *Veillonellaceae*, *Prevotellaceae*, *Bacteroidaceae*, *Coriobacteriaceae*	**Families**: *Lachnospiraceae*, *Ruminococcaceae*, *Enterobacteriaceae*	*Bacteroidaceae* OTU172, *Streptococcaceae* OTU834, *Ruminococcaceae* OTU181 and two *Lachnospiraceae* OTU477 and 629 were positively correlated with PANSS. *Veillonellaceae* OTU191 and *Ruminococcaceae* OTU725 were negatively correlated with PANSS
^L^**Study group**: Schizophrenia patients (*n* = 84) and healthy controls (*n* = 84) **Mean age (s.d.) shotgun group**: SSD: 35 (11); HC: 34(9) **Mean age (s.d.) 16S rRNA group**: SSD: 35 (11); HC: 35(11) **Sex (M/F) shotgun group**: SSD: 20/20; HC: 20/20 **Sex (M/F) 16S rRNA group**: SSD: 28/16; HC: 21/16 **PANSS (s.d.)**: 71.87 (1.85) **Experimental method**: shotgun metagenome sequencing or 16S rRNA gene sequencing from faecal samples	*α*: Chao 1 was significantly lower in the SSD group *β*: Different at the species level (NMDS)	**Phylum**: *Actinobacteria* **Class**: *Deltaproteobacteria* **Order**: *Actinomycetales*, *Sphingomonadales* **Families**: *Sphingomonadaceae* **Genus**: *Eggerthella*, *Megasphaera* **Species**: *Akkermansia muciniphila*, *Bifidobacterium adolescentis*, *Clostridium perfingens*, *Lactobacillus gasseri*, *Megasphaera elsdenii*	**Order**: *Rhodocyclales* **Families**: *Alcaligenaceae*, *Enterococcaceae*, *Leuconostocaceae*, *Rhodocyclaceae*, *Rikenellaceae* **Genus**: *Enterococcus*	Not reported
^M^**Study group**: Schizophrenia patients (*n* = 25) and healthy controls (*n* = 25) **Mean age (s.d.)**: HC: 54.7 (10.7); SSD: 52.9 (11.2) **Sex (F/M)**: HC: 15/10; SSD: 14/11 **SAPS (s.d.) and SANS (s.d.)**: 4.56 (3.3) and 4.16(5.1) **Experimental method**: 16S rRNA gene sequencing from faecal samples	*α*: No differences (Shannon index) *β*: Different (unweighted UniFrac, Bray–Curtis dissimilarity), with tighter clustering in HC (unweighted UniFrac, Bray–Curtis dissimilarity)	**Genera**: *Anaerococcus*	**Phylum**: *Proteobacteria* **Genera**: *Haemophilus*, *Sutterella*, *Clostridium*	*Bacteroides* was correlated with greater severity of depressive symptoms (*r* = 0.70, *p* = 0.0002). Decreased abundance of *Ruminococcaceae* was associated with increased negative symptoms (*r* = −0.74, *p* = 0.0002)
^N^**Study group**: Schizophrenia patients (*n* = 64) and healthy controls (*n* = 53) **Mean age (s.d.)**: HC: 39 (14); SSD: 42 (11) **Sex (F/M)**: HC: 18/35; SSD: 28/36 **Experimental method**: 16S rRNA gene sequencing from faecal samples	*α*: No differences (Shannon index, Simpson, ACE, Chao 1) *β*: Different (unweighted UniFrac, PCoA), with tighter clustering in HC (unweighted UniFrac)	**Phyla**: *Proteobacteria* **Classes**: *Gammaproteobacteria* **Orders**: *Aeromonadales*, *Fusobacteriales* **Families**: *Veillonellaceae*, *Prevotellaceae*, *Enterobacteriaceae*, *Succinivibrionaceae*, *Fusobacteriaceae*, *Lactobacillaceae* **Genera**: *Succinivibrio*, *Megasphaera*, *Collinsella*, *Clostridium*, *Klebsiella*, *Methanobrevibacter*, *Prevotella*, *Lactobacillus*, *Fusobacterium*, *Citrobacter*, *Acidaminococcus*, *Desulfovibrio*, *Phascolarctobacterium* **Species**: *Collinsella aerofaciens*, *Bacteroides fragilis*, *Prevotella stercorea*, *Lactobacillus mucosae*, *Bifidobacterium adolescentis*	**Phyla**: *Firmicutes* **Classes**: *Clostridia* **Orders**: *Clostridiales* **Families**: *Lachnospiraceae*, *Alcaligenaceae* **Genera**: *Blautia*, *Coprococcus*, *Roseburia*, *Streptococcus* **Species**: *Roseburia faecis*, *Blautia producta*, *Collinsella plebeius*, *Bacteroides eggerthii*	Not reported
^O^**Study group**: Schizophrenia patients (*n* = 19) and healthy controls (*n* = 69) **Mean age (s.d.)**: HC: 23.13 (3.89); SSD: 20.47 (4.57) **Sex (F/M)**: HC: 32/37; SSD: 4/15 **Positive symptom score (s.d.)**: 11.47 (6.76) **Negative symptom score (s.d.)**: 10.26 (5.13) **Disorganized symptom score (s.d.)**: 4.89 (4.48) **General symptom score (s.d.)**: 4.37 (3.52) **Experimental method**: 16S rRNA gene sequencing from faecal samples	*α*: No differences (observed OTUs, Shannon index) *β*: Different at the OTU level (PCoA)	**Orders**: *Clostridiales*, *Lactobacillales*, *Bacteroidales* **Genera**: *Lactobacillus*, *Prevotella* **Species**: *Lactobacillus ruminis*	Not reported	Not reported
^P^**Study group**: HC (*n* = 80); SSD (*n* = 80) **Mean age (s.d.)**: HC: 41.03 (14.34); SSD: 42.15 (13.13) **Sex (F/M)**: HC: 41/39; SSD: 36/46 **PANSS (s.d.)**: 59.12 (18.18) **Experimental method**: 16S rRNA gene sequencing from faecal samples	*α*: No differences (Faith's phylogenetic diversity, observed species, Shannon index) *β*: Different (PCoA)	**Phylum**: *Actinobacteria* **Genera**: *Collinsella*, *Corynebacterium*, *Lactobacillus*, *Mogibacterium*, *Succinivibrio*, *Undefined Eubacterium*, *Undefined Ruminococcus*	**Phylum**: *Firmicutes* **Genera**: *Adlercreutzia*, *Faecalibacterium*, *Ruminococcus*	PANSS scores were positively correlated with the abundance of the genus *Succinivibrio*. Increased negative symptoms were negatively correlated with the abundance of the genus *Corynebacterium*
^Q^**Study group**: First psychotic episode patients (*n* = 41) and healthy controls (*n* = 41) **Mean age (s.d.)**: HC: 24.7 (6.7); FPE: 23.1 (8.0) **Sex (F/M)**: HC: 21/20; FPE: 18/23 **PANSS-positive (s.d.)**: 22.4 (6.3) **PANSS-negative (s.d.)**: 22.3 (6.3) **PANSS-general (s.d.)**: 37.6 (7.4) **PANSS-total (s.d.)**: 82.3 (12.7) **Experimental method**: 16S rRNA gene sequencing from faecal samples	Not reported	**Species**: *Clostridium coccoides*	**Genera**: *Bifidobacterium*, *Lactobacillus* **Species**: *Escherichia coli*	Not reported
^R^**Study group**: Antipsychotic-free first episode patients (*n* = 90) and healthy controls (*n* = 81) **Mean age (s.d.)**: HC: 32.8 (12.3); FPE: 28.6 (9.54) **Sex (F/M)**: HC: 40/41; FPE: 44/46 **Experimental method**: shotgun metagenome sequencing	*α*: Greater in the SSD group at the genus level (Shannon index) *β*: Higher at the genus level in the SSD group (Bray–Curtis dissimilarity index)	**Genera**: *Acidaminococcus*, *Akkermansia*, *Anaerotruncus*, *Bifidobacterium*, *Citrobacter*, *Clavibacter*, *Comamonas*, *Coprobacillus*, *Cryptobacterium*, *Dialister*, *Enterococcus*, *Lactobacillus*, *Methanobrevibacter*, *Peptoniphilus*, *Pseudoflavonifractor*, *Veillonella*	**Genera**: *Butyrivibrio*, *Gemella*	Not reported
^S^**Study group**: First psychotic episode patients (*n* = 28) and healthy controls (*n* = 16) **Mean age (s.d.)**: HC: 27.8 (6); FPE: 25.9 (5.5) **Sex (F/M)**: HC: 8/8; FPE: 16/12 **Experimental method**: 16S rRNA gene sequencing from faecal samples	Not reported	**Families:** *Lactobacillaceae*, *Halothiobacillaceae*, *Brucellaceae*, *Micrococcineae* **Genera**: *Lactobacillus*, *Tropheryma*, *Halothiobacillus*, *Saccharophagus*, *Ochrobactrum*, *Deferribacter*, *Halorubrum*	**Families**: *Veillonellaceae* **Genera**: *Anabaena*, *Nitrosospira*, *Gallionella*	*Lachnospiraceae* (*r* = 0.38, *p* = 0.048), *Bacteroides* spp. (*r* = 0.38, *p* = 0.049) and *Lactobacillus* group (*r* = 0.48, *p* = 0.009) had positive correlations witch BPRS total scores. *Lactobacillus* group correlated positively with positive symptoms (*r* = 0.47, *p* = 0.012) *Lachnospiraceae* (*r* = 0.49, *p* = 0.008), *Ruminococcaceae* (*r* = 0.47, *p* = 0.011) and predominant bacteria (*r* = 0.42, *p* = 0.027) had positive correlations with negative symptoms
^X^**Study group**: Antipsychotic-free first episode patients (FSCZ, *n* = 40); chronically antipsychotic-treated patients (TSCZ, *n* = 85), healthy controls (*n* = 69) **Mean age (s.d.)**: HC: 23.14 (3.20); FSCZ and TSCZ: 24.19 (6.18) **Sex (F/M)**: HC: 32/37; FSCZ and TSCZ: 57/68 **Experimental method**: 16S rRNA gene sequencing from faecal samples	*α*: Richness (Chao) and diversity (Shannon index) was significantly higher in the HC compared to the TSCZ group. No differences between HC and FSCZ *β*: Unweighted UniFrac analysis demonstrated compositional differences across groups, weighted UniFrac analysis did not	**FSCZ – HC** **Family**: *Christensenellaceae*, *Enterobacteriaceae*, *Victivallaceae* **Genera**: *Escherichia* **TSCZ – HC** **Phyla:** *Proteobacteria* **Family**: *Christensenellaceae*, *Enterobacteriaceae*, *Enterococcaceae*, *Lactobacillaceae* **Genera**: *Escherichia*, *Bulleidia*, *Coprococcus*, *Trabulsiella*, *Enterococcus*, *Lactobacillus*, *Shigella*, *Streptococcus*, *Veillonella*	**FSCZ – HC** **Family**: *Pasteurellaceae*, *Turicibacteraceae*, *Peptostreptococcaceae*, *Veillonellaceae*, *Succinivibrionaceae* **Genera** : *Actinobacillus*, *Fusobacterium*, *Megasphaera*, *SMB53* **TSCZ – HC** **Phyla:** *Cyanobacteria* **Family**: *Pasteurellaceae*, *Turicibacteraceae* **Genera**: *Bacteroides*, *Parabacteroides*, *Turicibacter*	Not reported

HC, healthy controls; FPE, first psychotic episode patients; SSD, schizophrenia-spectrum disorder patients; FSCZ, antipsychotic-free first episode patients; TSCZ, chronically antipsychotic-treated patients; OTU, operational taxonomic unit; PLS-DA, partial least-squares discriminant analysis; NMDS, non-metric multidimensional scaling; PCoA, principal coordinates analysis; BPRS, Brief Psychiatric Rating Scale; PANSS, Positive and Negative Syndrome Score.

^K^Zheng et al. (2019); ^L^Xu et al. (2020); ^M^Nguyen et al. (2021); ^N^Shen et al. (2018); ^O^He et al. (2018); ^P^Li et al. (2020); ^Q^Yuan et al. (2018); ^R^Zhu *et al*. (2020b); ^S^Schwarz et al. (2018); ^X^Ma et al. (2020).

In the present review more studies reported inconsistent findings (*n* = 7) or no differences (*n* = 8) than studies who reported lower *α*-diversity in the psychiatric disorders (*n* = 5). These results are in line with other studies (Sanada et al., 2020; Simpson et al., 2021), suggesting that host–microbe interactions are more complex than can be modelled by *α*-diversity.

### Findings at different taxonomic levels

**Fig. 2.**
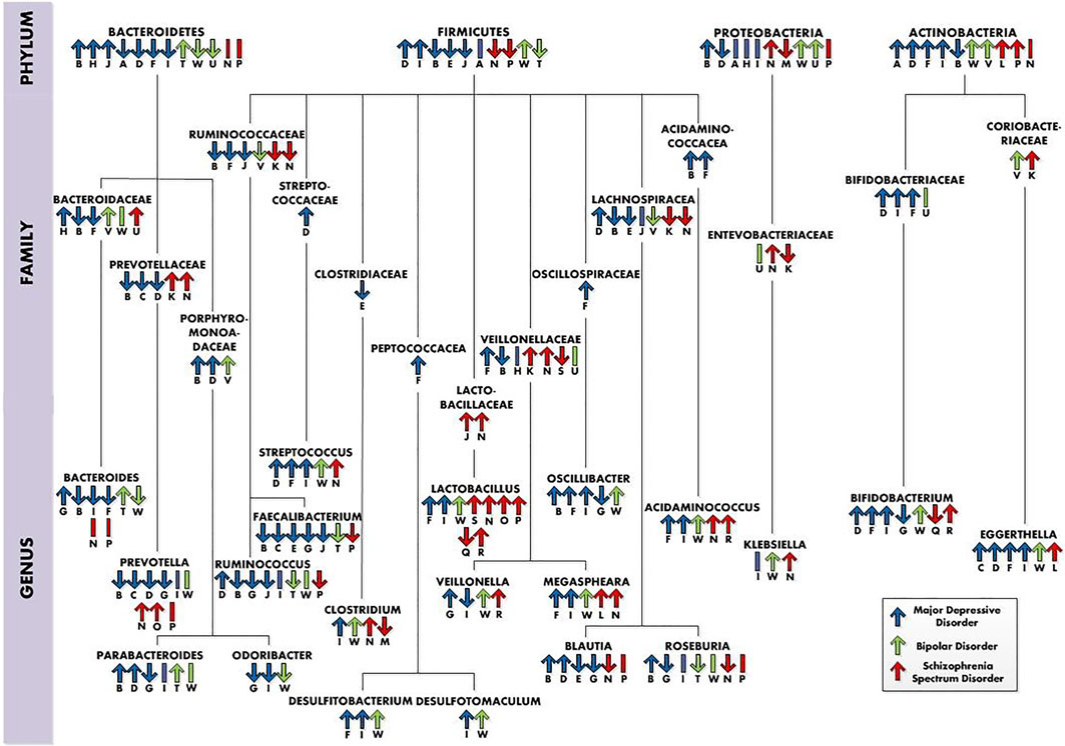
Taxonomic differences in neuropsychiatric disorders (at the phylum, family and genus levels), whereby ‘↑’ = higher relative abundance in the neuropsychiatric disorder group, ‘↓’ = lower relative abundance in the neuropsychiatric group and ‘I’ = no differences in abundance. The letters below the arrows refer to the studies the information was retrieved from and also can be connected to the letters in the tables. Studies: ^A^Zheng et al. (2016); ^B^Jiang et al. (2015); ^C^Kelly et al. (2016); ^D^Chung et al. (2019); ^E^Huang et al. (2018); ^F^Lai et al. (2021); ^G^Liu et al. (2016); ^H^Zheng et al. (2020); ^I^Rong et al. (2019); ^J^Liu et al. (2020); ^K^Zheng et al. (2019); ^L^Xu et al. (2020); ^M^Nguyen et al. (2018); ^N^Shen et al. (2018); ^O^He et al. (2018); ^P^Li et al. (2020); ^Q^Rong et al. (2019); ^R^Painold et al. (2018); ^S^Schwarz et al. (2018); ^T^Hu et al. (2019); ^U^Zheng et al. (2020); ^V^Painold et al. (2018); ^W^Rong et al. (2019).

A large number of bacterial taxa were significantly different in their relative abundance between control and psychiatric groups [MDD groups (Table 2), BD groups (Table 3) and SSD groups (Table 4)]. Interestingly, multiple bacterial taxa abundances were similar between psychiatric disorders (Fig. 2). All investigations reported taxonomic differences between the neuropsychiatric disorders and healthy control groups; in the next paragraphs the most important differences, similarities and findings are discussed.

#### Firmicutes

The most abundant phylum in the human gut, *Firmicutes*, showed multiple inconsistencies at the phylum, family and genus levels (Fig. 2). At the genus level, divergent findings were reported for *Ruminococcus*, which was higher in one MDD study (Chung et al., 2019), but lower in three other MDD studies (Jiang et al., 2015; Liu et al., 2020, 2016), one BD study (Hu et al., 2019) and one SSD study (Li et al., 2020). In line with these results, inconsistent findings were also reported in relation to clinical features. One study reported that decreased relative abundance of *Ruminococcaceae* was associated with an increase in negative symptoms (Nguyen et al., 2018). Another study reported *Ruminococcaceae* OTU725 to be negatively correlated with symptom severity in SSD (Zheng et al., 2019). In contrast, one study found a positive correlation of *Ruminococcaceae* with negative symptoms (Schwarz et al., 2018). One MDD study found a positive correlation between *Ruminococcus* and symptom severity scores (Chung et al., 2019). Another MDD study found a negative association of *Ruminococcaceae* with symptom severity (Liu et al., 2020).

Moreover, at the genus level *Faecalibacterium* were decreased in five MDD studies (Huang et al., 2018; Jiang et al., 2015; Kelly et al., 2016; Liu et al., 2020, 2016), one BD study (Hu et al., 2019) and one SSD study (Li et al., 2020). In line with these results Jiang et al. (2015) found a negative correlation between *Faecalibacterium* and depressive symptom severity. Furthermore, Liu et al. (2020) observed reduced *Faecalibacterium* in MDD patients with more severe symptoms. Moreover, the recently published systematic review and meta-analysis of Nikolova et al. (2021) observed depleted levels of *Faecalibacterium* in BD, MDD and schizophrenia as well. *Faecalibacterium*, especially species *Faecalibacterium prausnitzii*, is usually considered a ‘good’ gut bacterium and is associated with positive healthy outcomes, and its depletion with negative healthy outcomes (Ferreira-Halder, de Faria, & Andrade, 2017; Gacesa et al., 2022). Furthermore, *F. prausnitzii* was negatively associated with MDD (Gacesa et al., 2022).

The genus *Streptococcus* was reported to be higher in three MDD studies (Chung et al., 2019; Lai et al., 2021; Rong et al., 2019), one BD study (Rong et al., 2019) and one SSD study (Shen et al., 2018). In line with these results, Zheng et al. (2019) reported a positive correlation between *Streptococcaceae* OTU834 and symptoms severity. At the family level, the relative abundance of *Lactobacillaceae* was reported to be higher in two SSD studies (Schwarz et al., 2018; Shen et al., 2018). Consistently, at the genus level the relative abundance of *Lactobacillus* was significantly higher in two MDD studies (Lai et al., 2021; Rong et al., 2019), in one BD study (Rong et al., 2019) and in four SSD studies (He et al., 2018; Li et al., 2020; Schwarz et al., 2018; Shen et al., 2018). In line with these results, general symptom severity and positive symptom severity were positively correlated with *Lactobacillus* (Schwarz et al., 2018). These are interesting results since specific strains of *Lactobacillus* are commonly used in probiotics (Simpson et al., 2021). However, other studies have observed increased *Lactobacillus* as well in other disorders, like inflammatory bowel disease, indicating specific strains may have inflammatory potential (Wang et al., 2014). Rocha-Ramírez et al. (2017) found that several *Lactobacillus* species increased proinflammatory cytokines such as interleukin-8 (IL-8), tumour necrosis factor-*α*, IL-12p70 and IL-6. Moreover, Zhu *et al*. (2020b) reported increases of subspecies of *Lactobacillus* not typically present in the healthy gut in schizophrenia patients. It remains to be determined which species of *Lactobacillus* genus are increased in psychiatric disorders.

#### Actinobacteria

At the family level in the phylum Actinobacteria, *Coriobacteriaceae* were relatively more abundant in one BD study (Painold et al., 2018) and one SSD study (Zheng et al., 2019). From *Coriobacteriaceae*, the abundance of *Eggerthella* was relatively higher in four MDD studies (Chung et al., 2019; Kelly et al., 2016; Lai et al., 2021; Rong et al., 2019), in one BD study (Rong et al., 2019) and one SSD study (Xu et al., 2020). In line with these results, *Eggerthella* correlated positively with depression, anxiety and stress scores (Chung et al., 2019). Gacesa et al. (2022) found a positive association of the species *Eggerthella lenta* with BD. Moreover, Rekdal, Bess, Bisanz, Turnbaugh, and Balskus (2019) suggest that *E. lenta* is capable of selectively removing the *para*-hydroxyl group of dopamine, thereby decreasing dopamine levels. This suggestion of Rekdal et al. (2019) could explain the positive correlation found between *Eggerthella* and depression scores. Moreover, the BD patients in the study of Rong et al. (2019) were at the time of the study in a major depressive episode. *Eggerthella* could be related to depression in multiple disorders and could therefore be a target for depression. Unfortunately, *Eggerthella* and *E. lenta*, previously known as *Eubacterium lentum*, have been underrecognized due to historical difficulties with phenotypic identification, therefore not much information is available about the bacterial species.

Only a few studies have investigated microbiome difference among subtypes of psychiatric disorders. Hu et al. (2019) compared the GM of BD type 1 to BD type 2 patients with each other. They observed relatively higher relative abundance of the families *Streptococcaceae* and *Erysipelotrichaceae*, genera *Streptococcus*, *Bacilli* and *Veillonella* and lower relative abundance of the genus *Ruminococcus* in the BD type 1 group compared to the BD type 2 group. Zheng et al. (2020) compared unipolar depression to bipolar depression and found *Bacteroidaceae* and *Veilonellaceae* to be higher and *Enterobacteriaceae* and *Pseudomonadaceae* lower in MDD *v.* BD. Studying the GM of subtypes of psychiatric disorders has the potential to be of great value in understanding differences between these subtypes.

Inconsistencies across studies may be attributable partly to the heterogeneity in sample characteristics across studies. Most studies used small sample sizes; only one study used a sample size of more than 100 patients (Zheng et al., 2020). In addition, dietary habits may change among sites of study and socio-economic class of the participants. Then, psychiatric medication probably affects the microbiome. Methods used for obtaining the taxonomic profiles were not consistent. Moreover, statistical analysis used to compare GM composition between groups was quite heterogeneous across studies. Microbiota composition differences between groups were analysed by using several statistical methods, namely, analysis of composition of microbiomes, linear discriminant analysis effect size, Wilcoxon rank-sum test, Mann–Whitney *U* test, Kruskal–Wallis test and Welch's *t* test.

The most consistent findings across studies were higher relative abundances of the genera S*treptococcus*, *Lactobacillus* and *Eggerthella* and lower relative abundances of the genus *Faecalibacterium* in the neuropsychiatric disorders (Fig. 2), perhaps most interesting in the relationship between the psychiatric disorders and lower levels of *Faecalibacterium*. *F. prausnitzii* has been shown to have anti-inflammatory effects, to produce the SCFA butyrate and has been associated with improving the intestinal barrier by increasing levels of tight junction proteins occludin and E-cadherin (Carlsson et al., 2013; He, Zhao, & Li, 2021; Laval et al., 2015; Martín et al., 2015). Next to that, *F. prausnitzii* has been associated with smoking, which is in line with the fact that the percentage of smokers is higher in psychiatric disorders (Gacesa et al., 2022; Lê Cook et al., 2014). Multiple of the findings, like higher relative abundances of Actinobacteria and lower abundancy of *Prevotella* in MDD have been associated unhealthy dietary patterns. Higher relative abundances of Actinobacteria have been associated with high-fat and animal protein diet (Fritsch et al., 2021). Low carbohydrate intake has been associated with reduced *Prevotellaceae* (Kang et al., 2013). However, the majority of the studies did not control for diet, making it difficult to relate the findings to possible unhealthy diet patterns in the disorders.

## Treatments targeting the GM

Understanding how our GM can be targeted could lead to the development of microbiota-based therapies.

### Probiotics

Probiotics are defined as live organisms that exert a health benefit when ingested in an adequate amount. Probiotics contain living beneficial bacteria, traditionally from genera *Lactobacilli* and *Bifidobacteria*.

In MDD, Kazemi et al. (2019) conducted an 8 week randomized, double-blind, placebo-controlled study, in which 110 depressed patients were randomly assigned to receive probiotic (*L. helveticus* and *B. longum*), prebiotic (galactooligosaccharide) or placebo treatment. Probiotic supplementation resulted in a significant decrease in symptom severity compared to both the prebiotic and the placebo groups. Additionally, the serum kynurenine/tryptophan ratio was significantly decreased in the probiotic group compared to the placebo group. Another randomized, double-blind, placebo-controlled 8 weeks probiotics study by Akkasheh et al. (2016) studied a probiotic (*Lactobacillus acidophilus*, *Lactobacillus casei* and *Bifidobacterium bifidum*) in 40 MDD patients. As a result symptom severity decreased significantly in the probiotic group. In addition, serum insulin levels, insulin resistance and serum high-sensitivity C-reactive protein concentrations were decreased.

In BD, Dickerson et al. (2018) found that the adjunctive probiotic treatment (*Lactobacillus rhamnosus* strain GG and *Bifidobacterium animalis* subsp. *lactis* strain Bb12) for 24 weeks prevented rehospitalization in patients with acute mania (*n* = 66). Probiotic treatment also resulted in fewer days of hospitalization. Another study (*n* = 38) found no effects of probiotics compared to placebo on symptom severity of both depression and mania (Shahrbabaki et al., 2020). In another longitudinal cohort study 20 euthymic individuals with BD received a probiotic called ‘OMNi-BiOTiC^®^ Stress Repair’ (*B. bifidum* W23, *B. lactis* W51, *B. lactis* W52, *L. acidophilus* W22, *L. casei* W56, *Lactobacillus paracasei* W20, *L. plantarum* W62, *L. salivarius* W24, *L. lactis* W19) over a time period of 3 months (Reininghaus et al., 2020). Cognition concerning attention and psychomotor processing speed was improved as well as executive functioning.

Three studies investigated the effect of probiotics in SSD patients. One studied the combination three bacteria (*L. acidophilus*, *B. bifidum*, *L. reuteri* and *L. fermentum*) and vitamin D in a randomized placebo-controlled trial (*n* = 60) (Ghaderi et al., 2019). The combination was associated with significant improvement in symptom severity and had beneficial effects on metabolic profiles in reducing fasting plasma glucose levels, insulin concentrations, triglycerides and total cholesterol levels. Another study investigated a probiotic (*L. acidophilus*, *B. lactis*, *B. bifidum* and *B. longum*) and selenium co-supplementation in a randomized placebo-controlled trial with 60 people with chronic schizophrenia (Jamilian & Ghaderi, 2021). The probiotic and selenium co-supplementation resulted in a significant improvement in symptom severity compared to the placebo group. However, in both studies it is uncertain which component of the intervention was responsible for the changes. Only one study studied the effect of a probiotic alone (Dickerson et al., 2014). Their 65 participants were subjected to a double-blind adjunctive probiotic (*L. rhamnosus* strain GG and *B. animalis* subsp. *lactis* strain Bb12) or placebo therapy for 14 weeks. No significant differences were found in symptom severity between probiotic and placebo supplementation.

### Prebiotics

Prebiotics are non-digestible fibres that are selectively metabolized by the intestinal microbiome. Prebiotics mostly include fructans and glucans. So far, only one study has studied the effects of prebiotics in SSD, namely, by using the purified prebiotic Bimuno™ galactooligosaccharides (B-GOS^®^) formulation. Thirty-nine non-hospitalized participants with non-affective psychosis received either B-GOS^®^ or a placebo for 24 weeks. Participants performed the Brief Assessment of Cognition in Schizophrenia (BACS, Keefe et al., 2004) and the B-GOS^®^ group had significantly improved global cognitive performance, compared to the placebo group (Kao et al., 2019). While interesting, this study requires replication in larger studies.

### Faecal microbiota transplantation

Faecal transplants from depressed patients to germ-free rats show interesting results. Kelly et al. (2016) showed that FMT from depressed patients to microbiota-depleted rats can induce behavioural and physiological features characteristic of MDD in the receiving animals, including anhedonia and anxiety-like behaviour, as well as an increased plasma kynurenine/tryptophan ratio. In two studies, FMT from schizophrenia patients to germ-free mice led to hyperactivity and reduced anxiety in the open-field test (Zheng et al., 2019; Zhu et al., 2020a). Zhu et al. (2020a) also found that mice that received FMT from schizophrenia patients displayed impaired spatial learning and memory. Remarkable, however, was the heightened amount of social interaction these mice had compared to the control mice. In human, FMT has shown initial positive effects on autism spectrum disorder patients (Kang et al., 2019, 2017).

The most traditional and widely used probiotics (consisting of *Bifidobacterium* spp. and *Lactobacillus* spp.) are safe, yet they are not disease-specific. Although different *Lactobacillus* and *Bifidobacterium* strains were used in the probiotic studies mentioned above, most of the GM studies in the psychiatric disorders already showed heightened *Lactobacillus* and *Bifidobacterium* levels compared to their control groups. Rather, *F. prausnitzii* could be a promising target for therapeutic purpose in psychiatric patients (Verhoog et al., 2019). Research in rats showed preventive and therapeutic effects of *F. prausnitzii* on chronic unpredictable mild stress-induced depression-like and anxiety-like behaviour (Hao, Wang, Guo, & Liu, 2019). Supplementation of prebiotics consisting of inulin-oligofructose or inulin-type fructans and fructo-oligosaccharides have been demonstrated to increase *F. prausnitzii* levels (Dewulf et al., 2013; Hustoft et al., 2017; Ramirez-Fariaz et al., 2009), although this is not a consistent finding (Majid, Cole, Emery, & Whelan, 2014).

## Conclusions

The GM revolution has opened new frontiers for examining the relation between the brain and the GM in the context of understanding and treating/preventing psychiatric disorders. This paper provides a detailed overview of current findings regarding alterations of the GM in MDD, BD and SSD patients. All the reviewed studies reported alterations of the GM in the psychiatric disorders. Alterations may partly be caused by medication use, and other lifestyle factors like smoking, diet and alcohol use. Diversity metrics and microbial relative abundance reported to be anomalous across articles varied. The most consistent findings across studies were higher relative abundances of the genera *Streptococcus*, *Lactobacillus* and *Eggerthella* and lower relative abundance of the anti-inflammatory butyrate-producing genus *Faecalibacterium* in the psychiatric disorders. All three increased genera were reported to be associated with higher symptom severity. The similarities found between the disorders in the relative abundances and the associations of certain genera and symptoms suggest overlap in MDD, BD and SSD. So far, the results of probiotics trials have been highly discrepant, though few have shown promising results. Findings on prebiotics and FMT are too limited to draw definitive conclusions. There is a need of expanding our knowledge on bacterial species in bigger populations with psychiatric disorders, including the influence of medication and dietary habits. Information about differences in specific bacterial strains could lead to the use of disease-specific pro/prebiotics. In the end, study of the GM could lead to a new strategy of treating psychiatric disorders.
